# Endoscopic trans gastric assisted surgery for gastric tumors: Case report and description of a new surgical technique

**DOI:** 10.1016/j.ijscr.2019.11.049

**Published:** 2019-12-03

**Authors:** Jaime Solano, Manuel Cadena, Arturo Vergara, Luis Felipe Cabrera, Gabriel Herrera, Mauricio Pedraza

**Affiliations:** a Department of General Surgery, Fundación Santa Fe de Bogotá, Bogota, Colombia; b Department of Gastroenterology, Fundación Santa Fe de Bogotá, Bogota, Colombia; c Department of General Surgery, Universidad El Bosque, Bogota, Colombia; d Oncologist Surgeon, Memorial Sloan-Kettering Cancer Center, Fundación Santa Fe de Bogotá, Colombia; e Department of Medicine, Universidad El Bosque, Bogota, Colombia

**Keywords:** Gastric, Endoscopy, Surgery, Gastric tumors, Sub epithelial gastric lesion

## Abstract

**Background:**

Minimally invasive intragastric surgery [IGS] was first described by Ohashi in 1995 for early gastric cancer, with 3 trocars placed in the gastric lumen. Prior abdominal surgery is not a contraindication to IGS while the abdominal cavity is not explored, always that exist transilumination. But conversion to laparoscopic and open surgery can be challenging owing to the insufflated stomach and/or small bowel, although gas can be easily released via the gastrostomy.

**Case presentation:**

A 53-year-old female patient, presented with a sub epithelial gastric antrum lesion confirmed by endoscopic ultrasonography managed with surgical endoscopic percutaneous assisted transgastric technique [EPATS] using a gastrostomy tube and the endoscope.

**Discussion:**

We have been developing this operation since 2018. Nevertheless, we think EPATS is worthy to master, as PEIGS can salvage the entire stomach of patients with sub epithelial lesions in the lesser curve and in the esophagogastric junction, who otherwise would have to undergo total or proximal gastrectomy.

**Conclusion:**

We need to perform more cases for future comparative studies with percutaneous endoscopic intragastric surgery [PEIGS] in terms of parameters as pain, inflammation, complications, stenosis, oncological results and cosmesis.

## Highlights

• Laparoscopic intragastric surgery is a possible management for gastrointestinal storm tumors(GIST) treatment.
• EPATS as percutaneous endoscopic intragastric surgery (PEIGS) can salvage the entire stomach of patients with sub epithelial lesions.

## 1 Introduction

Minimally invasive intragastric surgery [IGS] was first described by Ohashi in 1995 for early gastric cancer, with 3 trocars placed in the gastric lumen. In 2011, Na et al. introduced single port intragastric surgery. In clinical trials, laparoscopic intragastric surgery with several trocars has been used to treat gastric gastrointestinal stromal tumors [GIST]. The largest series [n = 59] demonstrated a 29-month cumulative disease-free survival rate of 96.6 % [1].

Intragastric single incision with placement of a single port have risk of postoperative pain and port site herniation, similar to single-incision laparoscopic surgery. Incedence of bleeding reported in the largest series of intragastric surgery was 1.6 % [1 patient] [1,2].

Prior abdominal surgery is not a contraindication to IGS while the abdominal cavity is not explored, always that exist transilumination. But conversion to laparoscopic and open surgery can be challenging owing to the insufflated stomach and/or small bowel, although gas can be easily released via the gastrostomy. For that reason we develop a new surgical endoscopic percutaneous assisted transgastric technique [EPATS] for the resection of gastric sub epithelial lesions using only a gastrostomy tube and the endoscope [[1], [2], [3]]. This work has been reported in line with the SCARE criteria [13].

## 2 Case report

**Fig. 1:**
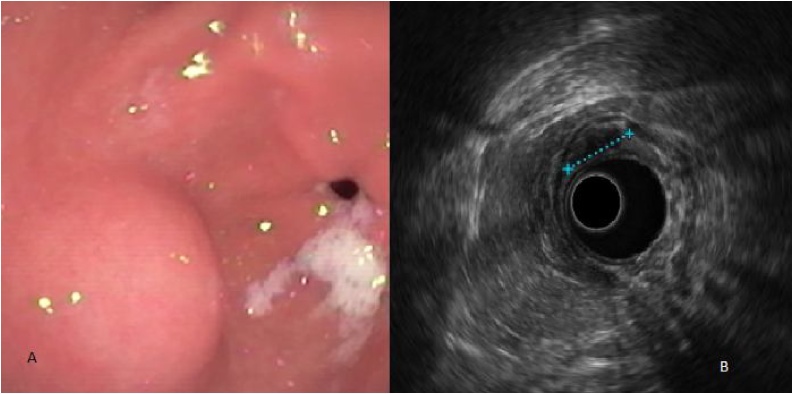
Sub epithelial gastric antrum lesion [**A**] in the second ultrasonographic layer of 25 mm [**B**].

A 53-year-old female patient, presented with a sub epithelial gastric antrum lesion in the second ultrasonographic layer of 25 mm confirmed by endoscopic ultrasonography (Fig. 1). History of abdominal pain, mainly localized in the left quadrant, associated with anorexia. The patient do not have previous abdominal surgery in her medical record. Abdominal computed tomography with negative lymph nodes and no other intra-abdominal conditions. The patient was taken to endoscopic percutaneous assisted transgastric surgery [EPATS] with no complications. Average surgical time of 58 min and minimal intraoperative bleeding. One day of hospital stay. Adequate tolerance of diet. The gastrostomy tube was extracted at the 3 week of the procedure with no complications. Final pathology showed a very low risk gastrointestinal stromal tumor [GIST] of the second ultrasonographic layer with 25 mm size and less than 5 mitosis.

## 3 Surgical technique

Under general anesthesia, the patient is placed supine. The surgeon stands at the right site of the patient for handle the endoscopic forceps through the gastrostomy tube and the gastroenterologist stands at the head of the patient for manage the endoscope. The scrub nurse stands on the left side of the patient.

**Fig. 2:**
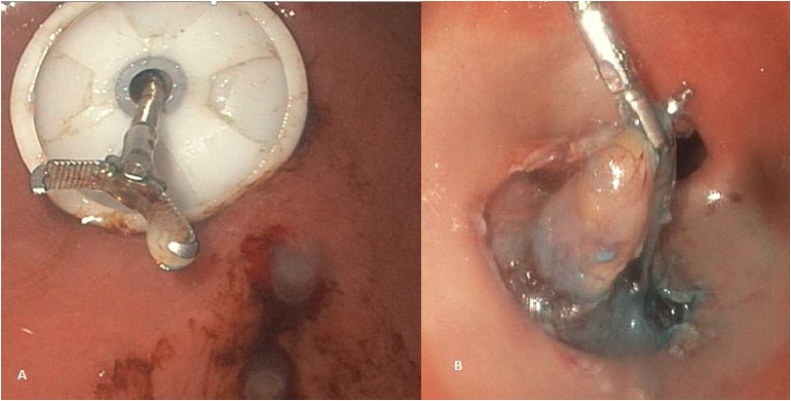
Percutaneous endoscopic gastrostomy is made [**A**] and use endoscopic biopsy - foreign body extraction forceps through the gastrostomy to do active traction of the gastric lesion [**B**].

**Fig. 3:**
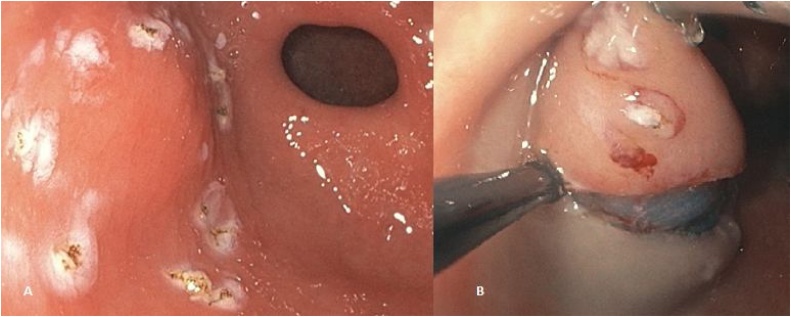
The resection line is marked by coagulation dots. Saline is then injected into the submucosal layer [**A**]. Resection is started with cutting the mucosal layer with a high-frequency hook through the endoscope [**B**].

**Fig. 4:**
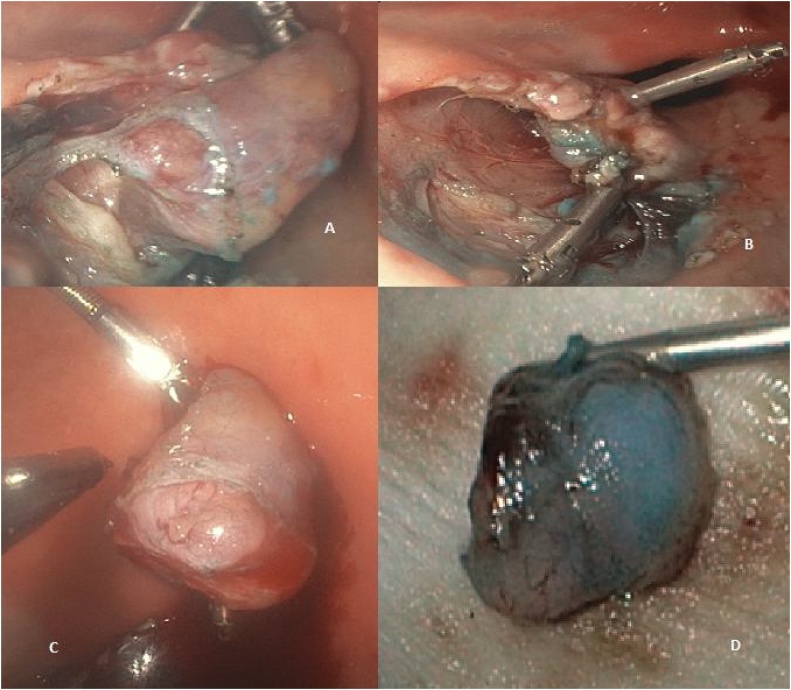
A grasping forceps [endoscopic biopsy and foreign body extraction forceps] is inserted through the gastrostomy tube to retract the mucosa [**A**]. The normal muscle bundles around the tumor are meticulously dissected with a high-frequency hook [**B**]. The excised specimen is extracted via the esophageal–oral route [**C** and **D**].

A percutaneous endoscopic gastrostomy is made in the upper abdomen, cranial to the umbilicus and to the left of the midline. A conventional gastrostomy tube of 18 French is placed in the stomach, and a pneumogastrium is created by carbon dioxide insufflation through the endoscope. The gastric lumen is insufflated with CO2 gas at 8–10 mmHg. We use endoscopic biopsy and foreign body extraction forceps through the gastrostomy to do active traction of the gastric lesion and allow the endoscopic knife perform an en bloc resection with no difficulty (Fig. 2). The surgeon’s position depends on the type of IGS procedure, while the screen is placed visa-vis to the surgeon and another screen for the gastroenterologist. The resection line is marked by coagulation dots. Saline is then injected into the submucosal layer. Resection is started with cutting the mucosal layer with a high-frequency hook through the endoscope (Fig. 3). A grasping forceps [endoscopic biopsy and foreign body extraction forceps] is inserted through the gastrostomy tube to retract the mucosa. When the mucosa is cut enough and the submucosa is dissected, the tumor surface [pseudocapsule] is clearly identified. The normal muscle bundles around the tumor are meticulously dissected with a high-frequency hook. The excised specimen is extracted via the esophageal–oral route (Fig. 4). After the endoscopic percutaneous assisted transgastric surgery [EPATS] is completed, the gastrostomy tube is extract at the 3 week of the procedure.

## 4 Postoperative management

All patients received intravenous infusion with antibiotics administration previous to the procedure and for 1 day after. Proton-pump inhibitor was injected for 24 h, and it was taken per orally for 6 weeks thereafter. The patients started clear fluid diet on postoperative day 1. All patients were discharge at postoperative day 1 if no abdominal pain and an adequate tolerance of diet. The gastrostomy tube was extracted at the 3 week of the procedure.

## 5 Discussion

Percutaneous endoscopic intragastric surgery [PEIGS] reported by Kanehira et al. [4], performing laparoscopic intragastric surgery with several trocars to treat gastric GIST, showed En-bloc enucleation, negative margins without tumor rupture in all patients with an average operation time of 172.3 min compared to our case with 58 min. 3 postoperative complications [one localized peritonitis, one bleeding, and one surgical site infection]. Average tumor size was 35.6 mm, higher than our case. The survival rate was 100 % with a disease-free rate of 98.3 % at 12 months and 96.6 % at 29 months with a follow-up period of 101 months. Confirming that PEIGS seems to be a curative procedure as other aggressive resection methods such as proximal gastrectomy as endoscopic percutaneous assisted transgastric surgery [EPATS] [4].

Intragastric single-port surgery [IGS] is performed by means of exteriorization of the gastric wall through the abdominal wall and placement of a single-port device with intragastric access. It differs from conventional laparoscopic or single-port surgery by the intragastric approach with direct endoluminal visualization of possible tumors and enables intragastric procedures but with EPATS we avoid the need of exteriorization and opening of the gastric wall. Plus a lower cost due to no need of single port device. Intragastric single-port surgery can be used in obese patients but might not overcome the surgical limitations in super-obese patients [body mass index [BMI] >50] but with EPATS we bypass this limitation [1,[5], [6], [7], [8]].

In general, for gastric sub epithelial lesions, wedge resection with safety margin is performed with a laparoscopic stapling device without need of lymphadenectomy. But when this lesions localized in the esophagogastric junction or in the lesser gastric curve, the patient’s needs a total or subtotal gastrectomy. For avoid this type of major gastric resections, endoluminal approaches can be an option to solve that problem. Transgastric surgery or so called endoluminal gastric surgery are based in the common concept of bring an endoscope and surgical instruments into the gastric lumen percutaneously, with some technical varieties. Hiki et al. [5] invented a combined method with per oral endoscopic and laparoscopic approach, which is called laparoscopic and endoscopic cooperative surgery [LECS]. We develop a new surgical technique the endoscopic percutaneous assisted transgastric technique [EPATS] performing an endoscopic resection with percutaneous traction through a gastrostomy tube, making the procedure more easy and feasible. The enucleation with EPATS would be the only way to preserve the entire stomach and achieve a complete removal of this type of lesions avoiding the need of open the gastric wall, gastric wedge resections or gastrectomy. Obtaining highly magnified endoscopic view to accurately identify structures in the soft tissue around the tumor, such as normal muscle bands, soft connective tissue in the submucosa, and small caliber vessels. By identifying and differentiating them from the pseudocapsule plus the transgastric traction, the endoscopic cutting procedure can be precisely advanced no matter how irregular the tumor configuration its. Our resection technique with a hook-shaped endoscopic electrocautery have been effective to avoid tumor rupture and allows the surgeon and gastroenterologist to sense the characteristic softness of the normal gastric muscle. EPATS procedure did not need hand-sew suture technique with interrupted suture in a radial pattern, showing a less surgical time and avoiding the risk of postoperative stenosis. Our patient no present postoperative complications. This type of endoscopic intragastric techniques like EPATS, are not recommend for lesions expanding to the esophagus higher than 2 cm from z-line or for exophytic tumors. Experts limit this types of techniques to tumor diameter of 5 cm or less, due to the increase risk of tumor rupture. Our long term patient outcome seems acceptable compared with other reports, secondary to the fact that tumor could be enucleated en-bloc without rupture of the capsule tumor, especially because pathological examination revealed a negative margin with a very low risk gastrointestinal stromal tumor [GIST] of the second ultrasonographic layer with 25 mm size and less than 5 mitosis, no need of adjuvant therapy with imatinib. Offering a chance to preserve the stomach, with no limitations of BMI or previous surgical interventions if gastric transillumination is achieved, been a preferable option in carefully selected patients with sub epithelial lesions or GISTs, when is performed by a skilled laparoendoscopic surgeon [4,[9], [10], [11], [12]].

## 6 Conclusion

We cannot recommend EPATS with just one case, for any gastroenterologist or surgeon. We have been developing this operation since 2018. Nevertheless, we think EPATS is worthy to master, as percutaneous endoscopic intragastric surgery [PEIGS] can salvage the entire stomach of patients with sub epithelial lesions in the lesser curve and in the esophagogastric junction, who otherwise would have to undergo total or proximal gastrectomy. We need to perform more cases for future comparative studies with PEIGS in terms of parameters as pain, inflammation, complications, stenosis, oncological results and cosmesis.

## Funding

Nothing to declare.

## Ethical approval

The study is exempt from ethnical approval in our institution.

## Consent

Written informed consent was obtained from the patient for publication of this case report.

## Author contribution

Dr. Solano, Dr Cadena and Dr Vergara: Evaluation and post-operative managementof the case along with surgical assistance.

Dr. Cabrera, Dr Cadena and Dr Herrera: Performed the surgical technique.

Dr. Pedraza, Dr Cabrera: Assisted the surgical procedure.

## Registration of research studies

N/A.

## Guarantor

Mauricio Pedraza Ciro.

Mpedraza93@gmail.com.

## Provenance and peer review

Not commissioned, externally peer-reviewed.

## Declaration of Competing Interest

Nothing to declare.
